# The taxanes: toxicity and quality of life considerations in advanced ovarian cancer

**DOI:** 10.1038/sj.bjc.6601496

**Published:** 2003-12-17

**Authors:** J P Guastalla III, V Diéras

**Affiliations:** 1 Department of Medical Oncology, Centre Léon Bérard, Lyon, France; 2 Department of Medical Oncology, Institut Curie, Paris, France

**Keywords:** ovarian cancer, paclitaxel, docetaxel, toxicity, quality of life

## Abstract

The taxanes paclitaxel and docetaxel show good activity in the management of advanced ovarian cancer when used in conjunction with platinum agents. Accumulating evidence from clinical studies, particularly the latest results from the phase III comparative SCOTROC study, indicates that the two drugs confer similar rates of tumour response and survival in women with this condition. However, it is clear that paclitaxel and docetaxel differ in their tolerability profiles and in other respects, and cannot be regarded as directly equivalent drugs. In particular, paclitaxel is associated with significant neurotoxicity; peripheral neuropathy has also been reported with docetaxel, but to a lesser extent. Neutropenia appears more prevalent with docetaxel than with paclitaxel, although clinical trial data show that this adverse effect is manageable and need not compromise dose delivery. Docetaxel is also associated with potential benefits accruing from shorter infusion times and lack of need for premedication with intravenous histamine H_1_ and H_2_ antagonists. Emerging quality of life data are expected to shed further light on the overall benefit of chemotherapy in women with advanced ovarian cancer in general, and on taxane−platinum combinations in particular.

Despite advances in diagnostic techniques and therapy, cancer of the ovary kills more women than any other tumour of the reproductive system and therefore remains the gynaecological malignancy of greatest concern in industrialised countries (National Library of Medicine, 1994; Dunton, 1997). Only a minority of patients present early enough for complete removal of the tumour to be successful, and chemotherapy is therefore the mainstay of treatment for the majority of women with ovarian cancer (Lister-Sharp *et al*, 2000) (80% of patients presented with advanced stage). Two classes of cytotoxic agents, the platinums and the taxanes, have emerged as key components of chemotherapy regimens for advanced disease (Kaye, 2001).

The place of paclitaxel in the treatment of advanced ovarian cancer is well established, but a newer member of the taxane group, docetaxel, has been developed more recently. Docetaxel differs from paclitaxel in a number of respects and represents an alternative taxane with considerable promise and potential tolerability advantages in the management of ovarian cancer (Guastalla *et al*, 1999; Vasey *et al*, 1999; Gorbounova *et al*, 2000; Vasey *et al*, 2001; Vasey and the Scottish Gynaecological Cancer Trials Group, 2001). In cases where different drugs show similar survival benefit in advanced malignant disease, issues relating to toxicity and quality of life (QoL) become increasingly important.

Current data suggest that the taxanes paclitaxel and docetaxel have similar efficacy, but regimens based on either of these two drugs have shown potentially important differences in their toxicity profiles. As it is always important to balance risks and benefits when setting out treatment plans for individual patients, knowledge of the tolerability differences between the taxanes available is clearly necessary for informed treatment decisions to be made. As part of this debate, this article reviews the toxicity profiles of each taxane and discusses QoL issues affecting patients with ovarian cancer.

## 

### Clinical benefit of taxane-based chemotherapy

The clinical benefit in terms of median overall and progression-free survival of combining paclitaxel rather than an alkylating agent with a platinum compound in stage III–IV (McGuire *et al*, 1996) or stage IIb–IV (Piccart *et al*, 2000) disease has been shown in first-line studies in a total of 1057 women. Further data from three major studies in a total of 1798 patients show enhancement of this benefit via improved overall tolerability when cisplatin is replaced by carboplatin (Neijt *et al*, 2000; du Bois *et al*, 2003; Ozols *et al*, 2003).

Concerns over neurotoxicity when paclitaxel and cisplatin are used together have led to evaluation of the combination of docetaxel with cisplatin: three studies of this type of first-line therapy showed encouraging overall clinical response rates of 69–74% (Guastalla *et al*, 1999; Vasey *et al*, 1999; Gorbounova *et al*, 2000) Neurotoxicity of any grade was reported in 16 and 26% of patients in two of the studies (Guastalla *et al*, 1999; Gorbounova *et al*, 2000), and 23% of patients in the other trial experienced neurotoxicity of severity greater than grade 1 (Vasey *et al*, 1999).

In addition, the promise of similar antitumour activity with reduced toxicity (notably neurotoxicity, ototoxicity, nephrotoxicity and gastrointestinal toxicity) when carboplatin is used in place of cisplatin as a taxane partner has prompted researchers to explore combinations of docetaxel and carboplatin. A feasibility study of first-line therapy in 139 patients treated with a range of combination dosages of carboplatin plus docetaxel yielded an overall response rate of 66% and median progression-free survival of 16.6 months, with extremely low levels of neurotoxicity (Vasey *et al*, 2001). The efficacy and safety of 3-weekly docetaxel 70–75 mg m^−2^ with carboplatin to achieve an area under the plasma drug concentration *vs* time curve (AUC) of 5–6 mg ml^−1^ min^−1^ have subsequently been confirmed in a series of three phase II studies in a total of 66 patients (Meyer *et al*, 1999; Kolevska *et al*, 2001; Vorobiof *et al* (2003)). In addition, preliminary comparative data from the Scottish Gynaecological Cancer Trials Group's phase III SCOTROC study in 1077 chemotherapy-naïve patients have indicated that paclitaxel and docetaxel have comparable efficacy when either is combined with carboplatin, but that there are significant differences in the tolerability characteristics of the two regimens (Vasey and the Scottish Gynaecological Cancer Trials Group, 2001).

## DOSAGE AND ADMINISTRATION ISSUES

### Dosage and schedule

Both docetaxel and paclitaxel are usually administered once every 3 weeks. Most patients with ovarian cancer receive paclitaxel as a 3-h infusion, whereas docetaxel is given over 1 h. This shortened infusion time suggests a potential advantage in terms of patient convenience and other factors such as clinic time and resources for docetaxel.

Although the majority of clinical trials involving the taxanes have involved 3-weekly administration, weekly schedules have also been investigated. There is an underlying pharmacokinetic rationale for such regimens in that they may mimic continuous infusions and thereby increase cellular drug exposure (Boehnke Michaud *et al*, 2000); weekly regimens have also been linked with reduced levels of myelotoxicity and subsequent potential for optimisation of dose intensity. Phase I data obtained in 18 previously treated patients with relapsed advanced ovarian cancer showed attainment of 90.75% of planned dose intensity with weekly escalating doses of paclitaxel (40–100 mg m^−2^) (Fennelly *et al*, 1997). A 30% partial response rate was noted in 13 assessable patients, and there was no evidence of cumulative myelosuppression.

Weekly infusions of docetaxel were associated with minimal myelotoxicity in women with advanced refractory ovarian cancer (Hainsworth *et al*, 1998). Myelosuppression was not dose limiting from 20–52 mg m^−2^ weekly in 35 evaluable patients. Grade III leucopenia was noted in 14% of the patients, and there were no reports of grade IV leucopenia or grade III or IV thrombocytopenia or anaemia.

A phase II trial from Japan has evaluated weekly paclitaxel 80 mg m^−2^ plus carboplatin to AUC 1.5–2.0 in 17 patients, 14 of whom had advanced ovarian cancer (Kurihara *et al*, 2001). The overall response rate of 64.7% included patients who had received previous chemotherapy. Neutropenia of severity greater than grade III was reported in 29.4% of patients, and QoL as demonstrated by the European Organization for Research and Therapy of Cancer (EORTC) core QLQ-C30 questionnaire suggested that weekly chemotherapy might be better tolerated overall than a 3-weekly schedule. In addition, Markman *et al* (2002) have recently assessed the effect of increasing the dose intensity of paclitaxel by giving 80 mg m^−2^ each week to 53 patients who had failed to respond to a conventional 3-weekly regimen consisting of paclitaxel and a platinum agent. An overall objective response rate of 25% was achieved in this phase II study, and peripheral neuropathy of grade 2 severity or greater was observed in seven patients (13%) of whom four withdrew from the study.

### Premedication guidelines

Patients receiving taxane therapy require premedication to minimise the risk of hypersensitivity reactions. However, the premedication guidelines recommended for paclitaxel and docetaxel are markedly different (Boehnke Michaud *et al*, 2000). As indicated in Table 1

**Table 1:** Premedication guidelines for the taxanes

	**Paclitaxel**				**Docetaxel**
**Drugs**	**1 h**	**3 h**	**24 h**	**96 h**	**1 h**
Histamine H_1_ antagonist (diphenhydramine 50 mg)	IV prior	IV prior	IV prior	None	None
Histamine H_2_ antagonist (cimetidine 300 mg, ranitidine 50 mg, famotidine 20 mg)	IV prior	IV prior	IV prior	None	None
Dexamethasone	20 mg p.o. 12 and 6 h prior	20 mg p.o. 12 and 6 h prior	20 mg p.o. 12 and 6 h prior	None	8 mg twice daily × 3 days starting 24 h prior

, patients who receive paclitaxel require both intravenous histamine H_1_ and H_2_ antagonists in addition to oral corticosteroids before 1-, 3- or 24-h infusions, although there is some evidence that premedication is not needed before prolonged infusions (those exceeding 96 h). By contrast, the premedication regimen recommended for patients receiving docetaxel consists of 3 days' oral dexamethasone (8 mg twice daily).

Corticosteroid therapy is used in part to prevent hypersensitivity reactions, but major aims are to delay the onset and decrease the severity of fluid retention, and to decrease the frequency and severity of skin and nail changes (Boehnke Michaud *et al*, 2000). A 5-day premedication was originally recommended for patients being treated with docetaxel, but this was subsequently reduced to 3 days after the latter regimen was shown to be associated with similar rates of fluid retention and less mucositis and infection.

## ADVERSE EVENTS

### Haematological toxicity

The dose-limiting toxicity of both docetaxel and paclitaxel is neutropenia or, more specifically, febrile neutropenia (Boehnke Michaud *et al*, 2000). According to collated data from single-agent clinical trials (Aventis Pharmaceuticals Products Inc., 2002), docetaxel 100 mg m^−2^ over 1 h every 3 weeks is associated with severe neutropenia (<500 cells mm^−3^) in 75.4% of patients; the corresponding rate with paclitaxel (135–300 mg m^−2^ over 24 h) is reported to be 52% (Mead Johnson Oncology Products, 2000). At a dose of 250 mg m^−2^ over 24 h, paclitaxel is associated with an incidence of febrile neutropenia of 16–36%, with other incidences being reported with other infusion durations (Boehnke Michaud *et al*, 2000). The rate of febrile neutropenia in patients receiving docetaxel 100 mg m^−2^ every 3 weeks is reported to be 11% in patients with normal liver function (Aventis Pharmaceuticals Products Inc., 2002).

Neutropenia is not cumulative with either taxane, and leucocyte counts typically recover 21 days after administration in nearly all patients (Pazdur *et al*, 1993; Fulton and Spencer, 1996). In almost all patients receiving paclitaxel, white cell counts begin to fall 5–7 days after administration, with a nadir being reached between day 7 and day 14. This phenomenon is seen earlier in docetaxel recipients: white cell counts start to fall 4–6 days after administration, and a nadir is reached between day 6 and day 8.

The severity of neutropenia can be minimised by the prophylactic addition of colony-stimulating factor to the first course of taxane chemotherapy, or when necessary throughout the treatment cycle (Boehnke Michaud *et al*, 2000). Clinical practice in this respect varies, with decisions on the use of colony-stimulating factor being based on the regimen being used and types of patient involved, although guidelines (e.g. from the American Society of Clinical Oncology (Ozer *et al*, 2000) and European Society of Medical Oncology (ESMO, 2001)) are also available.

Grade IV neutropenia was reported in 75% of 139 patients with stage Ic–IV ovarian cancer who received 3-weekly docetaxel 60–85 mg m^−2^ plus carboplatin to AUC 5 or 6 in the recent dose-finding study described earlier in this review (Vasey *et al*, 2001). Preliminary results from the first phase III comparison of docetaxel (75 mg m^−2^ over 1 h) plus carboplatin to AUC 5 with paclitaxel (175 mg m^−2^ over 3 h) plus carboplatin (the Scottish Randomised Trial in Ovarian Cancer, or SCOTROC, study in 1077 patients with grade Ic–IV ovarian carcinoma) supported these findings by showing a significant difference between the two treatments in tolerability profiles (Vasey and the Scottish Gynaecological Cancer Trials Group, 2001). A higher proportion of patients in the docetaxel arm than in the paclitaxel arm experienced grade IV neutropenia (80 *vs* 55%), although this was reported by the authors not to have compromised dose delivery or patient safety. As already discussed, guidelines are in place to aid clinicians in the use of colony-stimulating factors in patients likely to be at risk of myelosuppression, and appropriate treatment of this type would be expected to minimise the incidence and severity of neutropenia in patients receiving docetaxel with carboplatin in future studies.

Of additional interest in this respect are the observations (reported earlier) of Hainsworth *et al* (1998), who showed no grade IV leucopenia in their study of weekly docetaxel in 35 patients with advanced ovarian cancer. This suggests that weekly schedules of docetaxel may offer a clinical alternative for the minimisation of myelosuppression.

### Neurotoxicity

Paclitaxel use is associated with neuropathy that is predominantly sensory. This adverse effect appears to be dose-related, and is usually observed 24–72 h after single doses exceeding 250 mg m^−2^ or after multiple doses of 135–200 mg m^−2^. Patients who have received previous treatment with neurotoxic drugs appear in particular to be predisposed to this reaction. The neuropathy typically manifests initially as a burning or tingling sensation in the glove and stocking areas, and can progress to motor weakness with continued administration of the drug (Pazdur *et al*, 1993).

**Figure 1:**
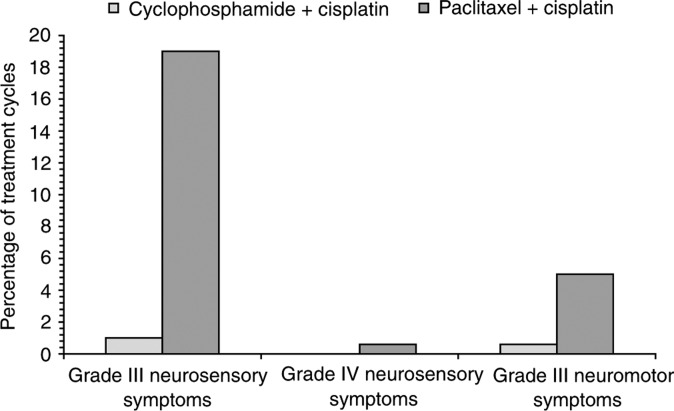
Neurosensory/neuromotor adverse events across all treatment cycles in 675 assessable patients with stage IIb–IV ovarian cancer in a phase III study (Piccart *et al*, 2000). Patients were randomly assigned to receive (i) paclitaxel 175 mg m^−2^ over 3 h followed by cisplatin 75 mg m^−2^ or (ii) cyclophosphamide 750 mg m^−2^ followed by cisplatin 75 mg m^−2^ for six to nine cycles.

The severity and frequency of peripheral neuropathy with paclitaxel may be related to infusion times in addition to dose, with more rapid infusions being associated with higher incidences. This is illustrated by results from two pivotal phase III studies that established the superiority of 3-weekly cisplatin plus paclitaxel over cisplatin plus cyclophosphamide in a total of 1057 evaluable patients with advanced ovarian cancer. Cisplatin and cyclophosphamide were given at the same dosages in both studies (75 and 750 mg m^−2^, respectively), but in one study a 24-h infusion of paclitaxel 135 mg m^−2^ was given (McGuire *et al*, 1996), whereas a 3-h infusion of 175 mg m^−2^ was used in the other (Piccart *et al*, 2000). There was no significant difference between treatments in incidence or severity of neuropathy in the 24-h infusion study (grade III–IV neurological symptoms in 4% of patients in each group); however, in the 3-h infusion study a marked neuropathic effect occurred in 19% of patients receiving cisplatin–paclitaxel compared with 1% of patients treated with cisplatin–cyclophosphamide (Figure 1

).

Concerns over the neurotoxicity of combinations of paclitaxel with carboplatin have been raised by several authors. Three key comparative trials of paclitaxel–cisplatin *vs* paclitaxel–carboplatin — the Dutch–Danish trial (Neijt *et al*, 2000), the Gynecologic Oncology Group (GOG) 158 study (Ozols *et al*, 2003) and the Arbeitsgemeinschaft Gynaekologische Onkologie (AGO) trial (du Bois *et al*, 2003) —reported similar efficacy for both combination regimens in patients with advanced ovarian cancer but less neurotoxicity with carboplatin than cisplatin. Nevertheless, as shown in Table 2

**Table 2:** Neurotoxicities reported in the Dutch–Danish and AGO studies of paclitaxel–cisplatin *vs* paclitaxel–carboplatin

	**Dutch–Danish (*n*=208)**		**AGO (*n*=772)**	**GOG 158 (*n*=792)**
**Treatment arm**	**Grade II neurotoxicity (%)**	**Grade III neurotoxicity (%)**	**Grade III–IV peripheral neuropathy (%)**	**Grade III neurotoxicity (%)**
Paclitaxel+cisplatin	26	6	14	8
Paclitaxel+carboplatin	17	3	7	7

a Paclitaxel 175 mg m^−2^ over 3 h+either cisplatin 75 mg m^–2^ or carboplatin to AUC 5 (Neijt *et al*, 2000).

b Paclitaxel 185 mg m^−2^ over 3 h+either cisplatin 75 mg m^−2^ or carboplatin to AUC 6 (du Bois *et al*, 2003).

, a noteworthy fraction of carboplatin recipients were affected by this adverse effect in the Dutch–Danish and AGO trials. According to the recent report of the GOG 158 trial (Ozols *et al*, 2003), 28% of patients who received carboplatin with paclitaxel (*n*=400) compared with 31% in the cisplatin–paclitaxel arm (*n*=392) were affected by grade II–IV neurotoxicity.

Peripheral neuropathy and myalgia/arthralgia have also been reported in patients receiving docetaxel, but to a lesser extent than in those receiving paclitaxel; neurotoxicity is also generally not dose limiting for docetaxel (Fulton and Spencer, 1996). Although fewer data are available for docetaxel than for paclitaxel in ovarian cancer, two studies of combination chemotherapy with docetaxel (dosages ranged from 40–85 mg m^−2^ every 3 weeks) and carboplatin (AUC 4–6) in a total of 189 patients with ovarian and other carcinomas both showed incidences of peripheral neuropathy (Markman *et al*, 2001) or grade II–III neurotoxicity (Vasey *et al*, 2001) of only 6%.

**Figure 2:**
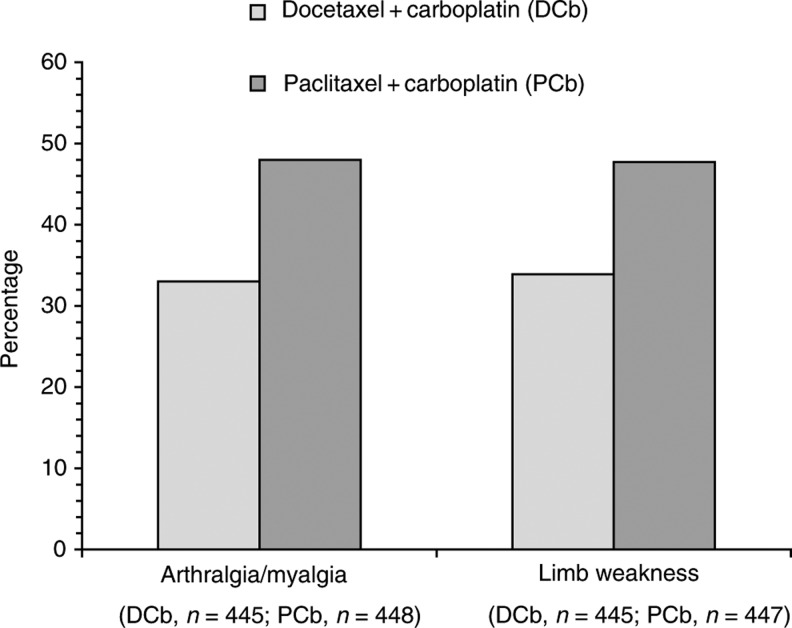
Incidences of arthralgic/myalgic symptoms and weakness in arms and legs reported in the multicentre phase III SCOTROC study of 3-weekly carboplatin to AUC 5 with either paclitaxel 175 mg m^−2^ infused over 3 h or docetaxel 75 mg m^−2^ over 1 h (Vasey and the Scottish Gynaecological Cancer Trials Group, 2002). Chemotherapy was given for up to six cycles as first-line treatment in women with grade Ic–IV ovarian cancer. Patient numbers denote those available for assessment of each adverse event shown.

Preliminary results from the SCOTROC study (Vasey and the Scottish Gynaecological Cancer Trials Group, 2001) supported these findings: grade II–III sensory neuropathy was reported in 10 and 28% of patients (*P*<0.001) in the docetaxel–carboplatin and paclitaxel–carboplatin groups, respectively. Most recent results (Vasey and the Scottish Gynaecological Cancer Trials Group, 2002) are similar: patients in the docetaxel arm reported less tingling in hands and feet and numbness in fingers and toes than those in the paclitaxel arm during treatment (*P*<0.001) and 6 (*P*<0.001) and 10 (*P*<0.005) months after randomisation. Arthralgia and myalgia were also reported significantly less frequently by docetaxel than paclitaxel recipients (Figure 2

). Note that these data were obtained with the EORTC's OV28 ovarian cancer module as described later in this review.

### Other adverse events

In general, proportions of patients experiencing grade III–IV nonhaematological adverse events such as arthralgia, myalgia, diarrhoea, hypersensitivity and fluid retention remain below 10% when premedication is administered with the taxanes as recommended (Boehnke Michaud *et al*, 2000; Kaye 2000; Vasey *et al*, 2001). Fluid retention syndrome appears to be confined to patients receiving docetaxel, although mild peripheral oedema has been reported with paclitaxel (Boehnke Michaud *et al*, 2000). The first sign of fluid retention is weight gain: early treatment with diuretics is effective in limiting the severity of the condition, which is most likely to be of concern in patients with congestive heart failure or other comorbid conditions (Boehnke Michaud *et al*, 2000).

As with the majority of chemotherapeutic agents, alopecia is common with either docetaxel or paclitaxel, with the whole body being affected approximately 10–14 days after the beginning of therapy (Boehnke Michaud *et al*, 2000). Although this adverse effect can have significant effects on body image, it does not pose any clinical risk to the patient.

### Quality of life

Since the definition over 50 years ago by the World Health Organization of health as not only the absence of disease and infirmity but also as the presence of physical, mental and social well-being, QoL issues have assumed increasing prominence in healthcare practice and research (Testa and Simonson, 1996). Accordingly, the goals of chemotherapy in advanced cancer should include improvement in QoL as well as increased duration of survival. Quality of life is therefore assuming ever-greater importance in the evaluation of cancer treatments.

Quality of life measurements and interpretation depend at least in part on patient characteristics, including stage and extent of disease, and on the type of treatment received, but there is general agreement on the usefulness of QoL assessments in the comparison of different therapies that confer similar survival in advanced cancer (Pignata *et al*, 2001). Alternatively, a clinically superior treatment may be so poorly tolerated that any survival advantage gained may not be sufficient to offset losses in QoL.

QoL issues of special relevance in patients with gynaecological (and therefore ovarian) cancer include limitations of sexual activity, early menopause, chemotherapy-induced toxicity and loss of body image (Pignata *et al*, 2001). Furthermore, it has been shown that awareness of severe disease is more frequent among women with ovarian cancer than among women with other types of malignancy (Porzio *et al*, 1999). Most generic QoL tools do not adequately capture disease-specific information or address treatment-related issues relevant to women with ovarian cancer, but the EORTC has recently developed an instrument specifically to deal with this problem.

### The EORTC OV28 module

The EORTC core QoL questionnaire QLQ-C30 was introduced in the early 1990s as a psychometrically robust and cross-culturally acceptable questionnaire designed to be applicable to a broad spectrum of cancer patients, and is now in widespread use worldwide. The development of OV28 as a QoL instrument for patients with ovarian cancer follows the EORTC's strategy of supplementing the generic QLQ-C30 questionnaire with disease- and/or treatment-specific modules to address issues of relevance to particular patient groups (Cull *et al*, 2001).

The provisional module as described in 2001 (Cull *et al*, 2001) has 28 items assessing abdominal symptoms, peripheral neuropathy, other chemotherapy-related side effects, hormonal symptoms, body image, attitude to disease and treatment and sexual functioning. The first 24 items of the module (items 31–54 as added to QLQ-C30), excluding questions on sexual functioning, have been assessed in a scaling analysis carried out with data from 277 patients participating in the SCOTROC trial (Cull *et al*, 2001; Vasey and the Scottish Gynaecological Cancer Trials Group, 2001, 2002). The mean scale scores were found to discriminate between trial patients before and after starting chemotherapy, and the OV28 module was indicated as a promising tool for the assessment of QoL of women with ovarian cancer. Emerging data from the SCOTROC study (Vasey and the Scottish Gynaecological Cancer Trials Group, 2002) show further the utility of OV28, particularly in terms of effect of chemotherapy on well-being (see neurotoxicity discussion earlier and Figure 2).

Quality of life has been investigated in patients with ovarian cancer in various studies, some of which are summarized in Table 3

**Table 3:** Examples of QoL assessments undertaken in studies in women with advanced (unless stated otherwise) ovarian cancer

**Study**	**Drugs given (no. of patients)**	**QoL instruments**	**Summary of findings**
Bodurka-Bevers *et al* (2000)	Various (246 – 74% with advanced disease)	Assessment of physical, functional, emotional and social/family well-being. CES-D, State Anxiety subscale of Spielberger State-Trait Anxiety Inventory, Zubrod scores	Performance status related to depression, anxiety and QoL problems, except for social well-being
Ersek *et al* (1997)	Various (study in 152 survivors – all disease stages)	QoL – Cancer Survivors tool	QoL moderately high despite negative findings related to facets of the illness and treatment experiences. Qualitative results reflect complexity of the cancer experience
Kornblith *et al* (1995)	Various (151 – 86% with advanced disease)	Memorial Pain Assessment Card; Memorial Symptom Assessment Scale; MHI; FLIC; KPS	QoL assessed at 3-month intervals. Impaired physical functioning (FLIC) most important predictor of psychological distress (MHI) at baseline. Significant differences in all QoL scales between patients with KPS⩽80 and those with KPS⩾90. Need for improved and more frequent assessment of psychological status stressed
Lakusta *et al* (2001)	First-line cisplatin and second-line carboplatin (chart review of 60 patients)	EORTC QLQ-C30	Less impact of carboplatin than cisplatin on QoL. Improved QoL over time in patients with recurrent disease supports palliative use of carboplatin
Schink *et al* (2001)	Paclitaxel+carboplatin (59)	FACT-OC	Improvement in QoL and physical well-being scores during this study of outpatient treatment
Willemse *et al* (1990)	Cyclophosphamide+doxorubicin+cisplatin (68)	TWiST	Correction of progression-free survival with TWiST suggested as being suitable for comparing effect of differing chemotherapy schedules on QoL
Willemse *et al* (1992)	Carboplatin+ cyclophosphamide (76)	TWiST	QoL as measured by TWiST significantly better than cyclophosphamide–doxorubicin–cisplatin (CAP-5) by comparison with historical control group (*n*=65)

*Abbreviations:* CES-D=Center for Epidemiologic Studies–Depression scale; EORTC QLQ-C30=European Organization for the Research and Treatment of Cancer Quality of Life Questionnaire core items; FACT-OC=Functional Assessment of Cancer Therapy – Ovarian Cancer scale; FLIC=Functional Living Index – Cancer; KPS=Karnofsky Performance Scale; MHI=Mental Health Inventory; QoL=quality of life; TWiST=time without symptoms of disease or treatment.

. A review of 20 papers, 10 of which were treatment-related QoL assessments and 10 of which dealt with other issues including psychometric evaluation, was published in the mid-1990s, and concluded that the Rotterdam Symptom Checklist and EORTC QLQ-C30 were the most appropriate instruments for use in patients with ovarian cancer at that time (Montazeri *et al*, 1996). However, this review was published before the development and introduction of the OV28 module. Thus, despite the inability of any instrument available to date to measure chemotherapy-related QoL directly, the lack of any comparative QoL data specific to patients receiving taxane therapy has resulted in considerable interest in the publication of the final analyses of the SCOTROC results.

### Psychological distress and QoL in ovarian cancer patients

As indicated in Table 3, studies are available to show the effect of psychological distress on patients with ovarian cancer. Kornblith *et al* (1995) examined physical, social and psychological well-being with a variety of instruments in 151 ovarian cancer patients (see Table 3), and reported significant psychological distress (expressed as 1.5 s.d. above a predetermined nationwide community sample standard in Mental Health Inventory psychological distress scores) in one-third of patients at study entry. Interestingly, but perhaps not surprisingly, impaired physical functioning was the most important predictor of heightened psychological distress.

A subsequent study evaluated psychological distress and QoL and examined the relationship between these problems and health and demographic variables in 246 patients with ovarian cancer who completed questionnaires (Bodurka-Bevers *et al*, 2000). Clinically significant depression and anxiety, assessed with the Center for Epidemiologic Studies Depression scale (CES-D) and State Anxiety subscale of the Spielberger State-Trait Anxiety Inventory, were found to be more prevalent than had been expected. Clinical depression was shown in 21% of patients, while 29% scored above the 75th percentile for anxiety. Further studies of screening and treatment of psychological distress were recommended to improve QoL outcomes in women with ovarian cancer.

Of particular interest in the context of this review is a recent study involving a chart review of 60 women with ovarian cancer undergoing chemotherapy with platinum agents (Lakusta *et al*, 2001). Analysis of EORTC QLQ-C30 questionnaire responses from these patients was used to relate biomedical variables to QoL outcomes and to compare patients receiving cisplatin as first-line therapy with those receiving palliative carboplatin for recurrent disease. Women receiving first-line cisplatin reported more appetite disturbance, diarrhoea and nausea than those on palliative carboplatin. Most notably, QoL declined over time in the newly diagnosed patients, whereas improvements were noted in those with recurrent disease, and lower QoL was found to predict death within 12 months of starting treatment. It was concluded on the basis of these results that the EORTC QLQ-C30 instrument can be used to test clinical assumptions and to influence treatment programmes in women with ovarian cancer undergoing chemotherapy. Furthermore, the findings appear to support the use of carboplatin as a palliative agent in advanced disease. They also indicate the potential utility of structured QoL assessments before clinic appointments as a means of improving overall patient care.

## CONCLUSIONS

Despite similarities in chemical structure and mode of action, docetaxel and paclitaxel cannot be regarded as having the same properties when used clinically. Study results have shown that the substitution of docetaxel for paclitaxel in a platinum-based doublet does not compromise efficacy, but that such substitution may confer benefits in terms of convenience to patients and toxicity. The reduced infusion time generally used with docetaxel (1 rather than 3 h), together with the need for premedication with oral dexamethasone only, is likely to be more convenient and to reduce the stress placed on patients by their treatment relative to paclitaxel. Docetaxel—carboplatin has also been associated with reduced frequency and severity of neurotoxicity relative to paclitaxel/carboplatin in clinical studies in patients with ovarian cancer. Higher incidences of neutropenia have been reported in patients receiving the docetaxel combination, but this is reported to be easily managed and is not associated with increases in rates of treatment discontinuation or death.

There are currently few data relevant to QoL in patients receiving taxane–platinum combinations for ovarian cancer, and QoL instruments cannot measure directly chemotherapy-related adverse effects. Nevertheless, the need for meaningful QoL assessments and the subsequent implications for treatment plans have become clear over the past decade, and results gained with the instruments available in comparative trials are awaited with interest. Familiarity of clinicians with differences between regimens in terms of toxicity, dosage and administration and QoL issues as data emerge and accumulate will assist in the optimisation of treatment decisions in patients with ovarian cancer.
